# Supplementary material for: Polyvinylidene Fluoride/Aromatic Hyperbranched Polyester of Third-Generation-Based Electrospun Nanofiber as a Self-Powered Triboelectric Nanogenerator for Wearable Energy Harvesting and Health Monitoring Applications
Source: Polymers (Basel). 2023 May 19;15(10):2375. doi: 10.3390/polym15102375 (PMC10224140; doi:10.3390/polym15102375)
Supplement: Supplementary file 1 [file polymers-15-02375-s001.zip › Supplementary Materials.pdf]

Supplementary Materials: Polyvinylidene Fluoride/Aromatic Hyperbranched Polyester of Third-Generation-Based Electrospun Nanofiber as a Self-Powered Triboelectric Nanogenerator for Wearable Energy Harvesting and Health Monitoring Applications

Ramadasu Gunasekhar [1], Ponnan Sathiyanathan [2,†], Mohammad Shamim Reza [2], Gajula Prasad [3], Arun Anand Prabu [1,*] and Hongdoo Kim [2,*]

[1] Department of Chemistry, School of Advanced Sciences, Vellore Institute of Technology, Vellore 632014, India
[2] Department of Advanced Materials Engineering for Information & Electronics, College of Engineering, Kyung Hee University, Yongin-si 17104, Gyeonggi-do, Republic of Korea
[3] School of Energy, Materials and Chemical Engineering, Korea University of Technology and Education, 1600, Cheonan-si 31253, Republic of Korea
* Correspondence: anandprabu@vit.ac.in (A.A.P.); hdkim@khu.ac.kr (H.K.)
† Present address: Department of Mechanical Engineering, Korea Advanced Institute of Science and Technology (KAIST), Daejeon 34141, Republic of Korea.

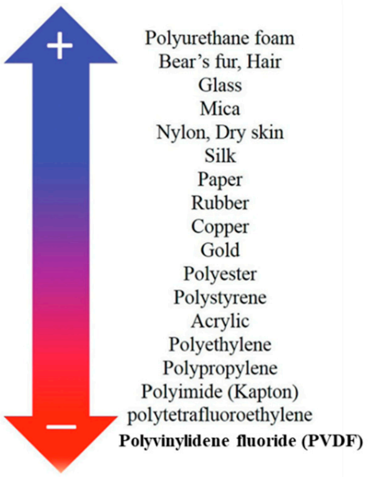

Figure S1. Triboelectric materials in series following a tendency to easily lose electrons (+) and to easily gain electrons (−).

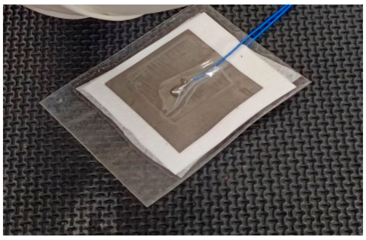

Figure S2: TENG device's optical view

Table S1: Draft comparison of triboelectric voltage from the literature data.

Reference	Materials used	Triboelectric Voltage
6	Ny 11 and PNy 11 films against EC5M, EC10M and EC5S, EC10S	50 to 270 V
13	ECF (P-60) against TPU	65 to 139 V
23	NR–CNF–AC	50 to 130 V
54	HPF-F	50 to 300 V

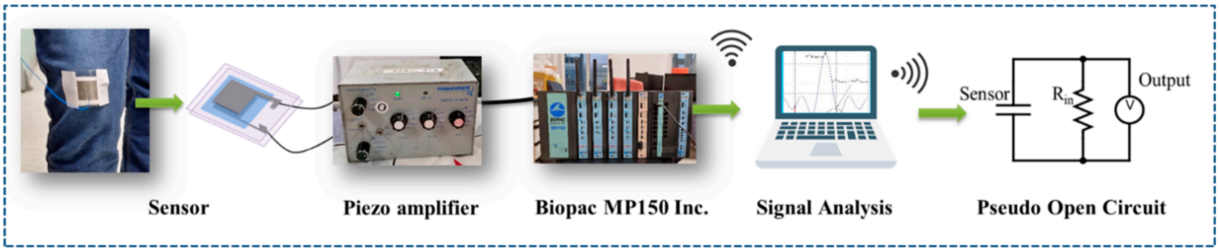

Figure S3: Schematic representation and general circuit for human health monitoring using TENG device.

Video S1: Demonstration of lighting 12 LEDs by open-circuit voltage of fabricated TENG.

Video S2: Demonstration of the sensitivity efficiency under various physical deformations of the sensor (tapping, twisting, bending, and folding).

Video S3: real-time measurement for the TENG pocket sensor.

Video S4: Demonstration of the TENG performance including walking and jumping when it is positioned on the shoe sole.
